# D_3_Creatine Dilution as a Direct, Non-invasive and Accurate Measurement of Muscle Mass for Aging Research

**DOI:** 10.1007/s00223-023-01124-w

**Published:** 2023-08-18

**Authors:** William J. Evans, Peggy M. Cawthon

**Affiliations:** 1 Department of Nutritional Sciences and Toxicology, University of California, Berkeley, Morgan Hall, Berkeley, CA USA; 2 Division of Geriatrics, Duke University Medical Center, Durham, NC USA; 3 California Pacific Medical Center Research Institute, San Francisco, CA USA; 4 University of California, San Francisco, CA USA

**Keywords:** Sarcopenia, Muscle mass, D3Creatine dilution, Sarcopenic obesity

## Abstract

Initial definitions of sarcopenia included the age-associated loss of skeletal muscle mass that was presumed to be associated with late-life reduced functional capacity, disability and loss of independence. Because no method for determination of muscle mass was available for large cohort studies of aging men and women, lean body mass determined by dual X-ray absorptiometry or bioelectrical impedance was used as a surrogate measure of muscle mass. The data from these studies showed either no or a poor relationship between LBM and functional capacity and health related outcomes, leading to the conclusion of many that the amount of muscle may not be associated with these age-associated outcomes. It was assumed that some undefined index of muscle quality is the critical contributor. These studies also consistently showed that muscle strength is lost more quickly than lean mass. Total body muscle mass can now be measured directly, accurately and non-invasively using the D_3_creatine (D_3_Cr) dilution method. D_3_Cr muscle mass, but not DXA derived LBM, is strongly associated with functional capacity, falls and insulin resistance in older men and women. In addition, D_3_Cr muscle mass is associated with risk of disability, hip fracture and mortality. New and emerging data demonstrate that low muscle mass may serve as a diagnostic criterion for sarcopenia.

Changing body composition is, perhaps, the most prominent and obvious feature of advancing age. Body fatness increases, even in men and women who remain weight stable as they grow older, the density of bones decrease, and the amount of skeletal muscle declines. More specifically, the geriatric syndrome, sarcopenia, was originally defined as the age-associated loss of skeletal muscle mass [1], that was hypothesized to increase the risk of functional decline and disability. Subsequent to the initial definition of sarcopenia, several (> 15) definitions have been published, many of which include lean body mass (LBM) rather than muscle mass [2–6]. However, an accurate assessment of whole-body muscle mass has proved to be elusive, and most investigators have used dual X-ray absorptiometry (DXA) or bioelectric impedance (BIA) assessments of lean body mass (LBM) as a surrogate measurement in large cohort studies. However, muscle mass is only one component of LBM that also includes body water, viscera, fibrotic and connective tissue. As a result, several cross-sectional and longitudinal aging cohort studies have reported little or no relationship between low LBM and increased risk of health-related outcomes including functional capacity, disability, and mortality [7–9]. A meta-analysis [10] of longitudinal observation studies in older people (≥ 65 year), conducted between 1976 and 2012 examined body composition (BIA, DXA, CT) and physical functional capacity. In the studies that examined lean mass (incorrectly described as “muscle mass”) the authors concluded that “low muscle mass was not significantly associated with functional decline”. They also concluded that the role of muscle mass in the development of functional decline was unclear but was “much smaller than the role of fat mass and muscle strength”. These conclusions that the loss of skeletal muscle mass, per se, is only weakly associated with functional outcomes in older people may be the result of using the measurement of lean mass as a proxy for muscle mass rather than using a direct measurement of muscle mass. Because LBM is so poorly associated with health-related outcomes in aging cohort studies, investigators have used DXA derived appendicular lean mass (ALM) to, perhaps, better approximate muscle mass. ALM is the sum of lean tissue in the arms and legs, typically expressed as ALM/ht^2^. While there is no disagreement that skeletal muscle is diminished with advancing age, the degree to which this reduction is associated with loss of functional capacity and risk of disability has not been established. In an effort to examine the existing data on the relationship between LBM and/or ALM and outcomes and publish a consensus definition of sarcopenia, a project was supported by the Foundation for the National Institutes of Health Biomarkers Consortium Sarcopenia (FINH). The group determined that strength (grip or quadriceps) but not lean mass was associated with mortality [11]. Although muscle mass was not measured in this study (only lean mass and thigh cross-sectional area from CT were assessed), the authors concluded that “Low *muscle mass* did not explain the strong association of strength with mortality, demonstrating that muscle strength as a marker of muscle quality is more important than quantity in estimating mortality risk”. Reductions in strength, but not lean mass, were associated with outcomes with advancing age which has led to the conclusion by several groups attempting to define sarcopenia to conclude that loss of muscle mass is poorly or unrelated to health-related outcomes and that the continuous decline in strength and functional capacity with advancing age must result from some undefined decrease in muscle quality.

## D_3_Creatine Dilution Method

Estimates of LBM do not provide an accurate measure of muscle mass for large cohort studies or randomized controlled trials. A suitable determination, particularly for cohort studies has proven to be elusive, until recently. Whole body magnetic resonance imaging is used to estimate total muscle volume; however, it is expensive, cumbersome, and impossible to use for some patient populations with contraindications (e.g., claustrophobia, non-MR compatible implanted metal or device). The D_3_creatine (D_3_Cr) dilution method measures the total body creatine pool size directly and takes advantage of well described features of creatine metabolism. Heymsfield et al. [12] estimated that about 98% (taken from Hunter [13]) of the body creatine pool is sequestered in the sarcomere; muscle has no capacity for creatine synthesis. Creatine is synthesized in the liver and kidney and transported against a huge concentration gradient into the sarcomere. The phosphorylation of creatine to high energy phosphocreatine represents an immediately available source of ATP for rapid muscle contraction. Because of its unique metabolic role for ATP synthesis, creatine and creatine phosphate are co-located with the contractile apparatus of muscle [14]. The method uses an oral tracer dose (30, 15 and 2 mg for adults, children, or infants) of deuterated creatine. It is 100% bioavailable and after entering the circulation, D_3_Cr is actively transported into all skeletal muscle cells. An additional aspect of creatine metabolism is that it is turned over through irreversible (in vivo) isomerization to creatinine which is rapidly excreted at a constant rate of about 1.7%/d. The enrichment of urine D_3_creatinine achieves isotopic steady state about 48 h and remains at steady state up to 96 h after ingestion of the tracer [15]. During this period of isotopic steady state, the enrichment of urine D_3_creatinine is the same as that of the intracellular enrichment of D_3_creatine, thus allowing a completely non-invasive sampling of the creatine pool. The method requires a fasting urine sample as consumption of foods with creatinine (mainly meat and meat products) results in increased urine creatinine excretion and dilution of the D_3_creatinine enrichment. Thus, the method requires knowledge of the tracer dose of D_3_Cr, ingestion of the dose, and collection of a single fasting urine sample 48 to 96 h later.

## Clinical Studies Using D_3_Cr Dilution

The method has been validated in rats and in adult humans using whole body MRI [15] and was further validated in premature infants cared for in a neonatal intensive care unit. In that study [16], longitudinal measurements of D_3_Cr muscle mass were made biweekly and revealed an astonishing accretion of muscle of about 90 g/wk (15%/wk). In boys with Duchenne muscular dystrophy (DMD), D_3_Cr muscle mass decreased with advancing age (ages 7–17 year) compared to age-matched, healthy controls who demonstrated growth related increases with advancing age. Remarkably, 3 DMD non-ambulant boys age 14–17 year had 13, 3 and 5% muscle mass relative to body weight [17]. These data demonstrate that muscle mass and changes in muscle mass can now be accurately and directly measured in populations and patients for which such assessments have not previously been available. D_3_Cr dilution method has been directly compared to DXA LBM in separate studies. In the initial validation study [15] in men and women between the ages of 19–84 year, DXA LBM vs D3Cr muscle mass were strongly related (*r* = 0.745); In 112 older men and women (70–95 year) the correlation was *r* = 0.6 [18]; in 74 older women (82.3 ± 5.4 year), *r* = 0.50 [19]; in 1382 older men (84.2), *r* = 0.66 [20]. Importantly, in each of these comparisons, the estimate of LBM by DXA is significantly and substantially higher than that of D_3_Cr muscle mass, often by more than 100%.

A criticism of the D_3_Cr dilution method is that its calculation of muscle mass relies on the assumption that the concentration of creatine is set at 4.3 g/kg wet weight of muscle, as the value of creatine pool size (in grams) that is estimated by the enrichment of D3Creatinine is divided by 4.3 to obtain total muscle mass in kg. The 4.3 g/kg wet weight is derived from rodent and humans [21, 22] and is based on an average estimated muscle fiber composition of 50% type I and II fibers. We note that type II, fast, fibers have a higher amount of creatine than type I, slow, fibers. Recently, Sagayama [23] et al. showed a strong relationship between total body MRI estimates of muscle mass vs D_3_Creatine muscle mass in young athletes. However, the use of 5.0 g/kg conversion factor for this population provided a better fit to estimate muscle mass in this population. These data demonstrate that creatine pool size may differ by muscle fiber composition, particularly in athletic populations. Some have called for use of creatine pool size estimated by D_3_Cr creatine dilution as the primary variable of interest to be used in analyses, rather than D_3_Cr muscle mass. We note that since D_3_Cr muscle mass (calculated using the set 4.3 g creatine/kg of wet weight muscle) is a monotonic transformation of the creatine pool size, effect estimates for standardized variables would be identical for either creatine pool size or D_3_Cr muscle mass (analyzed with or without adjustment for body size). For example, in the MrOS study, each standard deviation decrement in D_3_Cr muscle mass/body mass was associated with a 1.9-fold increased risk (hazard ratio, 1.9 (95%CI 1.2, 3.1) of incident ADL disability 2.2 years later; the same size association seen when this expressed as risk per SD decrement in creatine pool size/body mass (hazard ratio, 1.9 (95%CI 1.2, 3.1) [24].

The method requires that collection of a fasting urine sample during isotopic steady state (between 48 and 96 h after ingestion of the dose). Creatinine found in meat (and meat products) and fish can dilute the enrichment of D_3_Creatinine leading to an overestimation of creatine pool size and thus muscle mass. Typically, an overnight fast (after 10:00 PM) and use of the second voided urine sample prior to consumption of food greatly reduces to variability in the measurement due to dietary factors. Shankaran et al. [25] reported a CV of 3.1% for measurement of creatinine enrichment in urine sample collected three consecutive days. The method determines the dilution of the D_3_Cr dose in the body creatine pool. This requires absolute knowledge of the amount of the dose and no loss of the label before it is completely distributed throughout the body, including active transport into sarcomeres. However, some individuals “spill” a small amount of the D_3_Cr dose into urine [15]. This urine loss of tracer indicates that in some, the rate of endogenous production of creatine is greater than the rate of storage in muscle and, thus, a small amount of creatine is filtered in the kidneys and excreted. This small loss of label must be accounted for in the calculation of creatine pool size. Shankaran et al. [25] developed a correction algorithm based on the ratio of fasting urine creatine/creatinine in the sample collected for determination of D_3_creatinine enrichment. They also reported that most subjects are non-spillers and that the magnitude of correction for spillage of label is quite small.

## Osteoporotic Fractures in Men Cohort

The osteoporotic fractures in men (MrOS) Study is the largest study to date that has employed the D_3_Cr dilution method to assess muscle mass. In the study of ~ 1400 older men, low D_3_Cr muscle mass (but not low DXA lean mass or appendicular lean mass) was associated cross-sectionally with worse short physical performance battery (SPPB) score, slower walking speed, decreased lower extremity power, worse chair stand ability and greater likelihood of prevalent mobility limitations and disability [20]. The only muscle function related metric that was associated with both DXA appendicular lean mass and D_3_Cr muscle mass was grip strength. This population of older men (mean age of 84.2 year) reported multiple chronic diseases. Men in the higher quartiles of D_3_Cr muscle mass had significantly lower prevalence of type 2 diabetes, myocardial infarction, chronic heart failure and COPD. In addition, men in higher quartiles of D_3_Cr muscle mass/body mass generally had better cognitive function, had higher levels of physical activity, greater Life-Space and had markedly lower levels of exhaustion and fatigue. In longitudinal analyses over 3–5 years of follow-up, low D_3_Cr muscle mass was also associated with increased risk of hip fractures [26], injurious falls [20], incident disability and mortality [24]. Generally, the association of low D_3_Cr muscle mass with these outcomes was not explained by weakness or slow walking speed and there were few associations between low lean mass and these outcomes. In an accompanying commentary, Schaap [27] wrote, “In contrast to DXA, deuterated creatine (D_3_Creatine) assesses muscle mass directly” and “By isolating contractile muscle mass from noncontractile components including fat, the D_3_Creatine assessment is not only an accurate method to assess muscle mass but is less biased by obesity and aging than DXA ALM.”

**Fig. 1:**
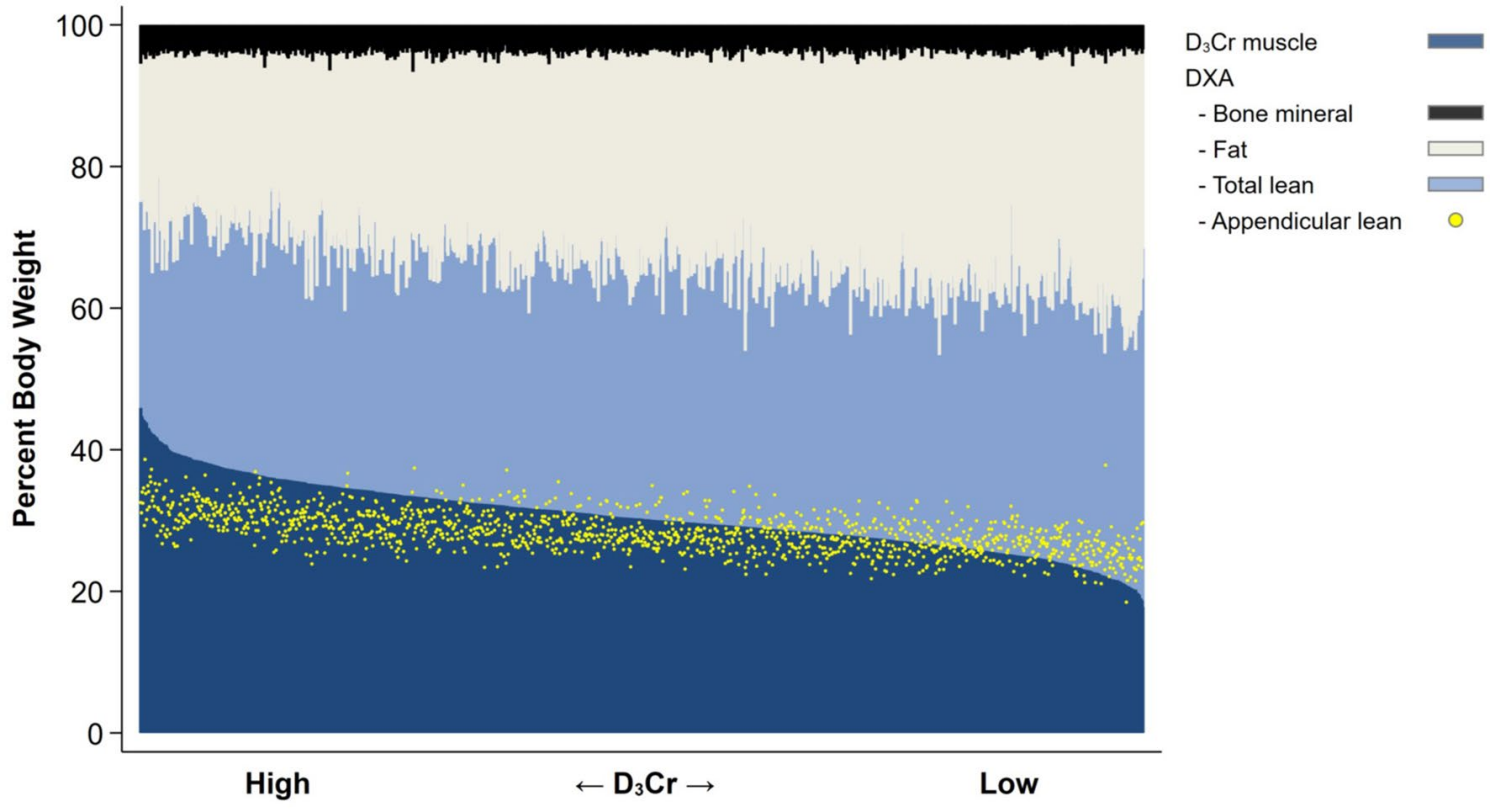
From Orwoll et al. [28]. Body composition using DXA and D_3_creatine dilution in 1376 older men. DXA provides an assessment of lean body mass (LBM, light blue + dark blue), fat mass, and bone mass while D_3_Cr is a measurement of muscle mass (dark blue). The men are ranked from highest muscle mass to lowest D3Cr muscle mass. Superimposed on the graph is DXA derived appendicular lean mass (ALM) in yellow. In this cohort, muscle mass was about 50% of LBM and was strongly associated with health-related outcomes including mortality, while LBM, ALM, or fat mass were not [20, 24, 26]

The association of sarcopenic obesity with outcomes was also examined in this cohort of older men [28]. Sarcopenic obesity is the combination of obesity with sarcopenia, a change in body composition typical of aging and the consensus of published literature has been that sarcopenic obesity poses even greater risks for poor health-related outcomes and disability than either obesity or sarcopenia alone [29]. However, when an accurate assessment of muscle mass using D_3_Cr dilution (rather than lean mass) was used, reduced muscle mass was strongly associated with important health-associated outcomes and the negative effects of adiposity were minimal, suggesting that obesity has little relevance for the understanding of important outcomes of sarcopenia and that sarcopenic obesity may be an anachronistic term that has few implications for physical function and risk of disability for older men. Figure 1 describes the body composition of the individual older men from this study by combining DXA with D3Cr muscle mass assessments [28]. Muscle mass is displayed from highest to lowest value and represents only about 50% of LBM. The light blue represents ‘residual’ non-muscle LBM consisting of body water, fibrotic, connective, visceral tissue, and smooth muscle.

However, before D_3_Cr muscle mass can be fully considered as a biomarker of sarcopenia, several limitations must be addressed. The MrOS cohort is not representative as it is comprised of very old men who mostly identify as Non-Hispanic White. However, two published studies from older women (from the Women’s Health Initiative cohort) show similar patterns in women [19, 30]. In community dwelling older women (mean age, 82.3 ± 5.4 year) D_3_Cr muscle mass was moderately related to DXA LM and ALM (*r* = 0.50 for both) with a significantly greater relationship between muscle mass and SPPB score compared to DXA LM or ALM [19]. In the same group of women, there was a significant inverse relationship between D_3_Cr muscle mass, and impaired insulin-glucose homeostasis [30].

Several new studies are underway to collect data from younger individuals, women and those who report other race and ethnic backgrounds, including projects in the Women’s Health Initiative life and longevity after cancer (LILAC) study, the Framingham Offspring Cohort, the Tobago Longitudinal Aging Study, the study of muscle mobility and aging (SOMMA), and others. In addition, there are limited data describing change in D_3_Cr muscle mass over time in adults. A study from MrOS in 40 participants (mean age 83.3 year) [31] found that the change in D_3_Cr muscle mass over 1.6 years is similar in magnitude to the change in grip strength and walking speed over the same period (with a mean loss of each of about 5%). Further, change in grip strength was significantly correlated with change in D_3_Cr muscle mass. During this time, there was no significant change in DXA lean mass, suggesting that the disconnect between declines in lean mass and declines in strength results from the poor measurement of muscle quantity by lean mass. While previous studies [32] have concluded that the age-associated decrease in strength is greater than the loss of muscle (using LBM measurements), these data strongly suggest that the loss of strength with age is closely linked to the loss of muscle mass. Additional data about change in D_3_Cr muscle mass from these ongoing studies will help characterize the change in muscle mass with aging and under other conditions.

Recent randomized controlled trials have demonstrated that the D_3_Cr is quite responsive to interventions that result in changes in muscle mass. Balachandran et al. [33] demonstrated that the D_3_Cr dilution method was sufficiently sensitive to detect changes in muscle mass in frail older subjects undergoing a resistance exercise training program compared to a non-intervention education program.

## Conclusion

The accurate determination of muscle mass in older men in the MrOS cohort and (so far) a limited number of older women from the WHI has provided insights into the importance of muscle rather than lean mass and its measurement. Although, these data are limited to largely white men and women at the present time, they reveal the previously unrecognized association between the amount of muscle and important health related outcomes and functional capacity in aging populations. In addition to its essential role for movement, skeletal muscle is the primary site of insulin stimulated glucose disposal, provides force to maintain bone density, principal component of age-associated decreased basal metabolic rate, and secretes myokines that exert autocrine and endocrine-like metabolic effects. The D_3_Cr dilution method can provide opportunities to examine how changing muscle mass may contribute to the increased risk of several age-associated diseases and syndromes. The strong and independent effects of D_3_Cr muscle mass to increased risk of instrumental activities of daily living (IADL) and mortality in the MrOS cohort suggest a more central role in changing cognitive function than previously thought. The use of lean body mass has resulted in a type 2 error for aging research that will continue to be propagated if LBM is considered a surrogate measurement of muscle mass. Zanker et al. [34, 35] used factor analysis of components of body composition in the MrOS cohort that were linked to poor mobility and determined that only D_3_Cr muscle mass factors were associated with negative outcomes. They concluded “these data support efforts to evaluate the D_3_Cr dilution method in clinical applications including in future definitions of sarcopenia”. In total, emerging data suggest that D_3_Cr muscle mass, perhaps even without assessment of strength or physical performance, could serve as a biomarker of sarcopenia in clinical or research settings that predicts adverse events.
